# Sobrevida de Pacientes com Insuficiência Cardíaca Aguda e Fração de Ejeção Intermediária em um País em Desenvolvimento – Estudo de Coorte no Sul do Brasil

**DOI:** 10.36660/abc.20190427

**Published:** 2021-01-27

**Authors:** Lucas Celia Petersen, Luiz Claudio Danzmann, Eduardo Bartholomay, Luiz Carlos Bodanese, Brenda Gonçalves Donay, Ellen Hettwer Magedanz, Adriana Vier Azevedo, Gustavo Farias Porciuncula, Marcelo Haertel Miglioranza

**Affiliations:** 1 Hospital São Lucas Porto AlegreRS Brasil Hospital São Lucas, Porto Alegre, RS - Brasil; 2 Hospital Universitário Universidade Luterana do Brasil CanoasRS Brasil Hospital Universitário da Universidade Luterana do Brasil, Canoas, RS - Brasil; 3 Hospital Moinhos de Vento Porto AlegreRS Brasil Hospital Moinhos de Vento, Porto Alegre, RS - Brasil; 4 Instituto de Cardiologia do Rio Grande do Sul Laboratório de Pesquisa e Inovação em Imagem Cardiovascular Porto AlegreRS Brasil Instituto de Cardiologia do Rio Grande do Sul - Laboratório de Pesquisa e Inovação em Imagem Cardiovascular, Porto Alegre, RS - Brasil; 5 Prevencor Hospital Mãe de Deus Porto AlegreRS Brasil Prevencor - Hospital Mãe de Deus, Porto Alegre, RS - Brasil

**Keywords:** Sobrevida, Insuficiência Cardíaca, Volume Sistólico, Prognóstico, Mortalidade, Adesão à Medicação, Epidemiologia

## Abstract

**Fundamento:**

A insuficiência cardíaca (IC) com fração de ejeção na faixa média ou intermediária (ICFEI) (em inglês, “ *mid-range ejection fraction* ) foi recentemente descrita em diretrizes europeia e brasileira recentes sobre o manejo da insuficiência cardíaca (IC). A fração de ejeção (FE) é um parâmetro importante para direcionar terapia e prognóstico. Estudos têm mostrado resultados conflitantes sem dados representativos de países em desenvolvimento.

**Objetivo:**

Analisar e comparar a taxa de sobrevida em pacientes com ICFEI com pacientes com IC e FE reduzida (ICFEr), e pacientes com IC e FE preservada, e avaliar as características clínicas desses pacientes.

**Métodos:**

Estudo coorte que incluiu pacientes com IC aguda admitidos no departamento de emergência de um hospital terciário, referência em cardiologia, localizado no sul do Brasil, entre 2009 e 2011. A amostra foi dividida em três grupos de acordo com a FE: reduzida, intermediária e preservada. Curva de Kaplan-Meier foi analisada de acordo com a FE, e uma análise de regressão logística foi realizada. A significância estatística foi estabelecida em p<0,05.

**Resultados:**

Um total de 380 pacientes foram analisados. A maioria dos pacientes apresentaram ICFEp (515), seguido de ICFEr (32%) e ICFEI (17%). Os pacientes com ICFEI apresentaram características intermediárias em relação à idade, pressão arterial, e diâmetros ventriculares, e a maioria era de etiologia isquêmica. O período mediano de acompanhamento foi de 4 anos. Não se observou diferença na sobrevida geral ou na mortalidade cardiovascular (p=0,03) entre os grupos de FE (FE reduzida: mortalidade de 40,5%; FE intermediária: 39,7%, e FE preservada 26%). A mortalidade hospitalar foi 7,6%.

**Conclusão:**

Não houve diferença na taxa de sobrevida entre os grupos de FE diferentes. Os pacientes com ICFEI apresentaram maior mortalidade por doenças cardiovasculares em comparação a pacientes com ICFEp. (Arq Bras Cardiol. 2021; 116(1):14-23)

## Introdução

A insuficiência cardíaca (IC) é uma síndrome complexa, considerada uma das principais causas de admissão hospitalar e morbimortalidade no mundo.^1 - 3^ Estudos observacionais descrevem taxas de mortalidade por IC de 4 a 12% durante internação hospitalar e de 20% a 30% um ano após a alta. As taxas de readmissão também são elevadas, variando de 20% a 30% em 90 dias, e atingindo 60% em um ano.^3 - 6^ Avanços na terapia cardiovascular têm sido associados a uma maior expectativa de vida e aumento na prevalência de IC na população idosa, criando a necessidade de um melhor conhecimento sobre a epidemiologia, o diagnóstico, e o tratamento dessa importante doença de saúde pública de países desenvolvidos e em desenvolvimento.

Apesar de a fração de ejeção (FE) não ser ideal para estratificação de pacientes, esse parâmetro tem sido historicamente utilizado na prática clínica para o direcionamento de terapias e estabelecimento de prognóstico.^7 , 8^ A fim de fomentar a pesquisa e melhor categorizar pacientes com IC, a Sociedade Europeia de Cardiologia ( *European Society of Cardiology* ) criou uma nova categoria de FE em sua recente diretriz sobre IC – a IC com FE na faixa média ou intermediária (ICFEI) (em inglês, “ *mid-range ejection fraction* ”)^9^ – que engloba pacientes com FE entre 40 e 49%.^1^ Essa nova classificação também foi adotada pela Sociedade Brasileira de Cardiologia pela diretriz de IC publicada em 2018.^3^ Desde então, muitos artigos descrevem os desfechos clínicos e as características da população com ICFEI, com resultados conflitantes. Enquanto alguns estudos com pacientes com IC aguda e crônica apresentaram sobrevida similar entre as três categorias de FE,^10 - 14^ outros apresentaram melhor sobrevida de ICFEI e IC com FE preservada (ICFEp) em comparação a pacientes com IC com FE reduzida (ICFEr).^15 , 16^

Dados sobre pacientes com ICFEI no Brasil e em países em desenvolvimento são escassos na literatura. O objetivo deste estudo foi analisar a sobrevida e as características clínicas de pacientes com ICFEI em comparação a pacientes admitidos com IC aguda e FE reduzida ou preservada.

## Métodos

### Delineamento e População do Estudo

**Figura 1:**
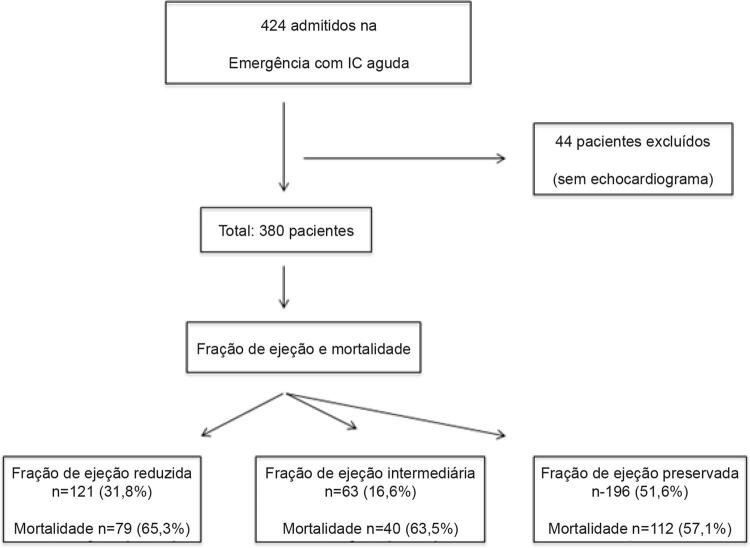
– Fluxograma da seleção da amostra do estudo; PAC: pressão arterial central; DM: diabetes mellitus; PH: pré-hipertensão; HT: hipertensão.

Este foi um estudo coorte prospectivo, derivado de um registro clínico de 424 pacientes consecutivos admitidos com IC aguda no departamento de emergência do Hospital São Lucas / Pontifícia Universidade Católica do Rio Grande do Sul, durante o período entre janeiro de 2009 e dezembro de 2011 ( Figura 1 ). Os critérios de inclusão foram: 1) idade maior que 18 anos; 2) diagnóstico de IC aguda definido pelos critérios de Framingham e posteriormente confirmado por ecocardiografia transtorácica. Os pacientes que não realizaram ecocardiografia durante a internação foram excluídos. O protocolo de registro clínico foi aprovado pelo comitê de ética em pesquisa do Hospital São Lucas (cidade de Porto Alegre) e um banco de dados de IC aguda foi construído. Os participantes assinaram um termo de consentimento.

O tamanho da amostra foi calculado com base na meta-análise *Meta-Analysis Global Group in Chronic Heart Failure* (MAGGIC) publicada em 2012. Para detectar uma diferença na mortalidade, seriam necessários entre 330 e 364 pacientes, com um poder de 80% e erro alfa de 5% (Roasoft e WinPepi Sample Size Calculator Software).

### Avaliação Clínica e Coleta de Dados

A avaliação clínica e o tratamento dos pacientes incluídos foram conduzidos pelo médico da emergência e a equipe de cardiologia de plantão, seguindo-se o protocolo de rotina da instituição, sem interferência dos pesquisadores. A coleta de dados foi realizada utilizando-se um formulário estruturado e revisão de prontuários médicos.

Os primeiros sinais e sintomas dos pacientes foram registrados na chegada ao departamento de emergência, antes da admissão, por avaliação clínica, hemodinâmica, dos sinais vitais e da classe funcional segundo a *New York Heart Association* . Além do tratamento prescrito durante a internação, os medicamentos usados em casa e prescritos na alta foram avaliados.

Causas da IC descompensada foram analisadas – isquemia miocárdica (se algum tipo de revascularização miocárdica foi realizada durante a internação hospitalar); hipertensão não controlada (no caso de hipertensão em estágio ≥II na chegada ao hospital); arritmia (ritmo não sinusal, exceto na presença de fibrilação atrial permanente com taxa ventricular controlada); baixa adesão ao tratamento medicamentoso; infecção (diagnóstico durante a internação).

A etiologia isquêmica da IC foi considerada na presença de revascularização do miocárdio prévia ou recente; teste funcional com isquemia maior que 10%; exame anatômico mostrando estenose maior que 50% na artéria coronária esquerda ou 70% na artéria descente anterior esquerda ou em outros dois vasos coronários. Também foram registrados comorbidades relatadas pelo paciente ou diagnosticadas durante internação.

Como parte do protocolo institucional, cada paciente foi submetido a um eletrocardiograma de 12 derivações, radiografia de tórax, exames laboratoriais (hemograma, eletrólitos, função renal, perfil lipídico, glicemia, urina), e um ecocardiograma transtorácico com medida de FE usando o método de Simpson.

A amostra foi dividida em três grupos de acordo com a medida de FE no ecocardiograma: reduzido (<40%), intermediário (40-49%), e preservada (≥ 50%). O diagnóstico de ICFEp foi feito de acordo com diretrizes existentes, baseado principalmente no diâmetro atrial, na massa do ventrículo esquerdo e na função diastólica.

### Acompanhamento e Desfechos

Os dados de desfechos foram obtidos por revisão de prontuário e pelo Sistema de Informações sobre Mortalidade do Centro de Informação de Saúde do Rio Grande do Sul para identificar mortalidade e causa de morte até dezembro de 2017.

Causa direta de morte foi estabelecida segundo Classificação Internacional de Doenças (10ª edição).

O desfecho primário avaliado foi mortalidade geral, e o desfecho secundário foi mortalidade por causas cardiovasculares (infarto agudo do miocárdio, IC, acidente vascular cerebral e arritmia).

### Análise Estatística

As variáveis contínuas com distribuição normal (analisada pelo teste de Kolmogorov-Smirnov) foram expressas como média e desvio padrão ou mediana e intervalo interquartil, conforme apropriado. Comparação entre variáveis categóricas foi realizada pelo teste do qui-quadrado. As curvas de sobrevida foram estimadas pelo método de Kaplan-Meier, utilizando-se o teste de log-rank para comparação entre as categorias de FE. Regressões logísticas univariada e bivariada foram avaliadas para determinar as principais variáveis relacionadas à mortalidade. Significância estatística foi definida como p<0,05. As análises estatísticas foram realizadas usando-se o programa Statistical Package for the Social Sciences (SPSS) Statistics, versão 21.0.0.

## Resultados

De 424 pacientes internados com IC aguda, 380 pacientes foram estudados ( Figura 1 ). A maioria dos pacientes apresentavam ICFEp (51,6%), seguido de ICFEr (31,8%) e ICFEI (16,6%). A idade média foi 68 ± 13 anos, e a maioria era do sexo feminino (53%). O tempo mediano de acompanhamento foi de 4,0 anos (intervalo interquartil: 0,92 – 7,62 anos).

### Características Clínicas

A população do estudo com ICFEp era predominantemente composta por mulheres de idade avançada, com pressão sanguínea mais alta e frequência cardíaca e dimensões do ventrículo esquerdo mais baixos. O grupo com ICEFr foi composto majoritariamente por homens jovens, com pressão sanguínea mais baixa e frequência cardíaca e dimensões do ventrículo esquerdo mais altos. Pacientes com ICEFI apresentaram características intermediárias entre os pacientes com ICEFp e ICEFr com relação à idade, sexo, pressão sanguínea, frequência cardíaca e dimensões do ventrículo esquerdo ( Tabelas 1 e 2 ).

**Tabela 1:** – Dados demográficos e comorbidades dos pacientes com insuficiência cardíaca categorizados segundo fração de ejeção

Características	Total	Fração de ejeção < 40%	Fração de ejeção 40-49%	Fração de ejeção ≥ 50%	p
	**% (N = 380)**	**31.8% (N=121)**	**16.6% (N=63)**	**51.6% (N=196)**	
**Demográficas**					
Idade média (anos)	68,1 ±13,8	64,0 ±12,6^b^	66,6 ±15,3^ab^	71,3 ±13,4^a^	<0,001
Sexo feminino	52,9% (201)	35,5%(43)^b^	52,4%(33)^ab^	63,8%(125)^a^	<0,001
Índice de massa corporal média (Kg/m^2^)	28,1 ±6,5	26,6 ±6,1	29,2 ±6,3	28,6 ±6,6	0,100
**Comorbidades**					
Etiologia isquêmica	40,0% (152)	46,3% (56)	52,4% (33)	32,1% (63)	0,004
Hipertensão	93,2% (354)	90,1% (109)	92,1% (58)	95,4% (187)	0,176
Dislipidemia	74,8% (243)	76,2% (80)	76,9% (40)	73,2% (123)	0,796
Doença renal crônica	46,2% (156)	42,1% (45)	57,9% (33)	44,8% (78)	0,135
Diabetes Mellitus	45,9% (169)	43,9% (50)	50,8% (32)	45,5% (87)	0,668
Valvulopatia	35,1% (99)	28,1% (25)	36,2% (17)	39,0% (57)	0,230
Doença pulmonar obstrutiva crônica	32,2% (111)	42,1%(45)	21,1% (12)	29,8% (54)	0,014
Dispositivo cardíaco implantável	20,7% (78)	27,3% (33)	24,6% (15)	15,5% (30)	0,031
Fibrilação atrial	20,0% (76)	5,8% (22)	2,1% (8)	12,1% (42)	0,085
Bloqueio do ramo esquerdo	16,3% (62)	7,1% (27)	2,9% (11)	6,3% (24)	0,133
Acidente Vascular cerebral	17,5% (62)	16,2% (18)	12,3% (7)	19,8% (37)	0,390
Hipotiroidismo	18,0% (49)	16,7% (14)	28,9% (13)	15,4% (22)	0,112
Uso excessivo de álcool	19,4% (67)	32,4% (34)	12,5% (7)	14,1% (26)	<0,001
Tabagismo	17,7% (63)	25,9% (29)	11,9% (7)	14,6% (27)	0,021
Câncer	12,0% (43)	12,4% (14)	3,4% (2)	14,5% (27)	0,070

*Análise estatística: teste de qui-quadrado com resíduo ajustado e análise de variância (ANOVA) com teste de Bonferroni (letras “a” e “b”).*

**Tabela 2:** – Dados clínicos, laboratoriais e de imagem na admissão

Características	Total	Fração de ejeção < 40%	Fração de ejeção 40-49%	Fração de ejeção ≥ 50%	p
	**% (N = 380)**	**31,8% (N=121)**	**16,6% (N=63)**	**51,6% (N=196)**	
**Demográficas**					
Pressão arterial sistólica média (mmHg)	140 (±35)	128 (±26)^b^	139 (±33)^ab^	147 (±39)^a^	<0,001
Frequência cardíaca média (bpm)	91 (±23)	96 (±22)^a^	89 (±20)^ab^	88 (±22)^b^	0,006
Hemoglobina (mg/mL)	12,0 (±2,6)	12,6 (±2,5)^a^	11,9 (±2,3)^ab^	11,6 (±2,6)^b^	0,004
Creatinina (mg/dL)	1,8 (±1,2)	1,9 (±1,5)	1,9 (±0,9)	1,8 (±1,2)	0,615
Ureia (mg/dL)	71 (±46)	70 (±48)	76 (±40)	71 (±50)	0,766
Sódio (mg/dL)	137 (±17)	139 (±4,4)	139 (±3,1)	137 (±2,5)	0,324
Potássio (mg/dL)	4,3 (±0,7)	4,4 (±0,8)^ab^	4,5 (±0,6)^a^	4,2 (±0,7)^b^	0,017
Diâmetro sistólico do ventrículo esquerdo (cm)	3,5 (±1,8)	5,0 (±1,6)^a^	4,0(±1,5)^b^	3,1 (±0,8)^c^	<0,001
Diâmetro diastólico do ventrículo esquerdo (cm)	4,7 (±2,0)	5,7 (±1,8)^a^	5,2 (±1,9)^b^	4,8 (±0,9)^b^	<0,001
Diâmetro do átrio esquerdo (cm)	3,9 (±1,7)	4,3 (±1,3)	4,0 (±1,5)	4,3 (±0,9)	0,182

*Teste ANOVA com teste de Bonferroni (letras “a”, “b”, e “c”).*

Nos pacientes com ICFEI, os níveis plasmáticos de potássio foram maiores na admissão, e a isquemia miocárdica foi a principal etiologia da IC ( Tabela 1 ). Os pacientes com ICEFI apresentaram uma menor prevalência de doença pulmonar obstrutiva crônica, tabagismo e consumo de álcool. Os pacientes com ICFEr apresentaram maior uso de inibidor de enzima conversora de angiotensina, antagonistas de mineralocorticoides, diuréticos de alça, e dispositivos aparelhos cardíacos eletrônicos implantáveis ( Tabelas 2 e 3 ). A maioria dos pacientes apresentaram um perfil hemodinâmico “úmido e quente” na admissão, sem diferença entre os grupos segundo FE.

**Tabela 3:** – Medicamentos usados pelos pacientes em casa

Medicamentos	Total	Fração de ejeção < 40%	Fração de ejeção 40-49%	Fração de ejeção ≥ 50%	p
	**% (N = 380)**	**31,8% (N=121)**	**16,6% (N=63)**	**51,6% (N=196)**	
Diurético de alça	60,1% (218)	67,0% (77)	66,7% (38)	53,9% (103)	0,043
Inibidor de enzima conversora de angiotensina	51,5% (187)	63,5% (73)	38,6% (22)	48,2% (92)	0,043
Betabloqueador	49,0% (179)	50,0% (58)	45,6% (26)	49,5% (95)	0,641
Ácido acetilsalicílico	40,7% (149)	44,0% (51)	45,6% (26)	37,3% (72)	0,367
Estatina	43,3 (156)	43,0% (49)	50,0% (28)	41,6% (79)	0,533
Digoxina	25,6% (93)	40,0% (46)	24,6% (14)	17,3% (33)	<0,001
Hipoglicemiante oral	20,9% (76)	19,1% (22)	17,5% (10)	23,0% (44)	0,568
Insulina	19,3% (70)	20,9% (24)	24,6% (14)	16,8% (32)	0,370
Antagonistas de mineralocorticoides	18,5% (67)	27,0% (31)	22,8% (13)	12,0% (23)	0,003
Bloqueador de canal de cálcio	16,9% (61)	8,8% (10)	15,8% (9)	22,1% (42)	0,011
Diurético tiazídico	14,6% (53)	14,0% (16)	14,0% (8)	15,2% (29)	0,954
Anticoagulante oral	14,0% (51)	14,7% (18)	10,5% (6)	14,1% (27)	0,660
Bloqueador de receptor de angiotensina	12,2% (44)	5,2% (6)	17,5% (10)	14,7% (28)	0,019

*Análise estatística: teste de qui-quadrado com resíduo ajustado.*

A baixa adesão à terapia medicamentosa foi a principal causa de IC descompensada, seguida por infecção em pacientes com ICEFr e ICEFp, respectivamente ( Tabela 4 ).

**Tabela 4:** – Causas da descompensação da insuficiência cardíaca

Causas	Total	Fração de ejeção < 40%	Fração de ejeção 40-49%	Fração de ejeção ≥ 50%	p
	**% (N = 380)**	**31.8% (N=121)**	**16.6% (N=63)**	**51.6% (N=196)**	
Medicamentos	30,5% (116)	42,1% (51)	27,0% (17)	24,5% (48)	0,003
Infecção	27,1% (103)	19,0% (23)	19,0% (12)	34,7% (68)	0,003
Arritmia	18,7% (71)	15,7% (19)	19,0% (12)	20,4% (40)	0,721
Hipertensão	14,5% (55)	9,1% (11)	15,9% (10)	17,3% (34)	0,120
Isquemia miocárdica	7,6% (29)	8,3% (10)	12,7% (8)	5,6% (11)	0,174
Sobrecarga de sal	7,4% (28)	7,4% (9)	7,9% (5)	7,1% (14)	0,978
Desconhecida	18,2% (69)	18,2% (22)	23,8% (15)	16,3% (32)	0,407

*Análise estatística: teste do qui-quadrado com resíduo ajustado.*

### Desfechos

**Figura 2:**
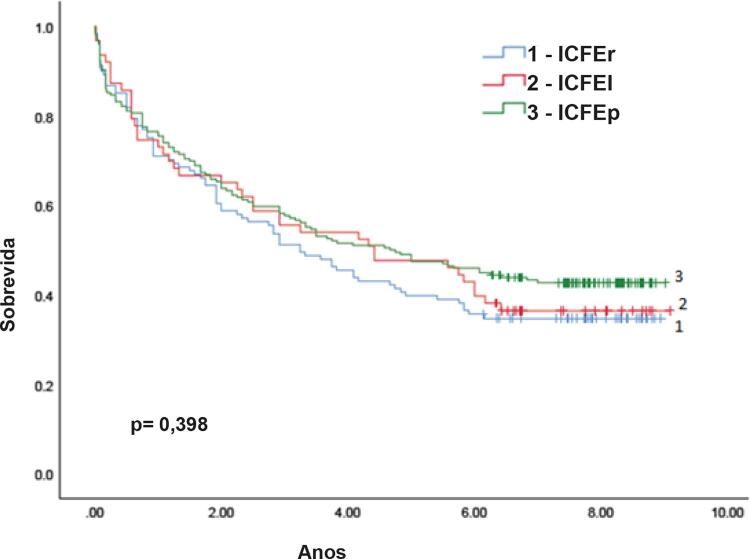
– Curva de sobrevida geral. ICFEr: insuficiência cardíaca com fração de ejeção reduzida; ICFEI: insuficiência cardíaca com fração de ejeção intermediária; ICFEp: insuficiência cardíaca com fração de ejeção preservada.

A mortalidade hospitalar foi de 7,6%. A mortalidade geral nos oito anos de acompanhamento foi de 60,7%, sem diferença estatística entre as categorias de FE ( Figura 2 ).

A mortalidade por grupo de FE, ao longo do período de acompanhamento, está descrita na Tabela 5 .

**Tabela 5:** – Mortalidade durante o período de acompanhamento

Mortalidade geral	Total	Fração de ejeção < 40%	Fração de ejeção 40-49%	Fração de ejeção ≥ 50%	p
	**% (N = 380)**	**31.8% (N=121)**	**16.6% (N=63)**	**51.6% (N=196)**	
Hospitalar	7,6% (29)	6,6% (8)	4,8% (3)	9,2% (18)	0,453
1 mês	10,8% (41)	10,7% (13)	7,9% (5)	11,7% (23)	0,699
3 meses	14,7% (56)	13,2% (16)	14,3% (9)	15,8% (31)	0,814
12 meses	26,6% (101)	28,5% (35)	27,0% (17)	25,0% (49)	0,742
5 meses	55,0% (209)	60,3% (73)	52,4% (33)	52,6% (103)	0,439
8 meses	60,7% (231)	65,3% (79)	63,5% (40)	57,1% (112)	0,398

*Análise estatística: teste ANOVA.*

**Figura 3:**
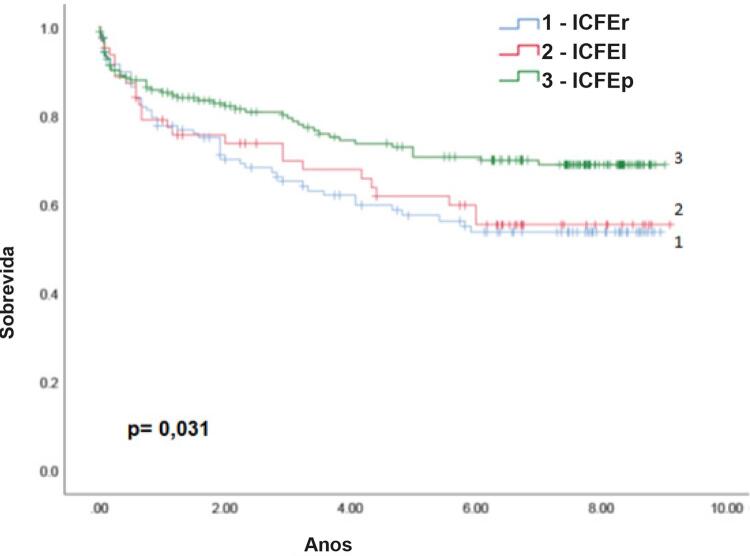
– Curva de sobrevida para doenças cardiovasculares, ICFEr: insuficiência cardíaca com fração de ejeção reduzida; ICFEI: insuficiência cardíaca com fração de ejeção intermediária; ICFEp: insuficiência cardíaca com fração de ejeção preservada.

A taxa de sobrevida média foi de 4,7 anos (IC 95%: 3,7 – 5,6), com tendência de aumento gradual com o aumento da FE (FE reduzida: 4,3 anos; FE intermediária: 4,7 anos; e FE preservada 4,9 anos). A mortalidade cardiovascular foi responsável por quase metade das mortes (54,1%). Houve uma diferença estatisticamente significativa entre os grupos de FE quando as mortes cardiovasculares foram analisadas separadamente (p=0,031): FE reduzida 40,5%; FE intermediária 39,7%; e FE preservada 26% ( Figura 3 ).

### Análise Univariada

Quando a regressão logística univariada foi analisada com variáveis categóricas, a presença de fibrilação atrial e níveis de ureia maior que 92 mg/dL foram identificados como fatores de risco. Quando analisada como uma variável contínua, valores mais altos da pressão sistólica foram identificados como um fator protetor. Os dados coletados na chegada a emergência são descritos na Tabela 6 .

**Tabela 6:** – Regressão logística univariada em relação à mortalidade geral

Regressão logística univariada	p	Odds Ratio	Intervalo de confiança de 95%
ICFEr	0,245	1,44	0,78 - 2,65
ICFEI	0,62	1,2	0,58 - 2,49
ICFEp	_	1	_
Etiologia isquêmica	0,775	1,07	0,66 - 1,74
Diâmetro diastólico > 5,6 cm	0,421	1,26	0,72 - 2,12
Pressão arterial sistólica < 115 mmHg	0,494	1,22	0,69 - 2,12
Pressão arterial sistólica	0,006	0,99	0,98 - 0,99
Creatinina > 2,75 mg/dl	0,741	1,15	0,51 - 2,58
Ureia > 92 mg/dl	0,034	2,00	1,05 - 3,80
Fibrilação atrial	0,028	1,98	1,08 - 3,64
Bloqueio do ramo esquerdo	0,921	1,03	0,54 - 1,97

*ICFEr: insuficiência cardíaca com fração de ejeção reduzida; ICFEI: insuficiência cardíaca com fração de ejeção intermediária; ICFEp: insuficiência cardíaca com fração de ejeção preservada*

### Análise Multivariada

A regressão logística multivariada revelou que não houve diferença quanto às características clínicas ou taxa de mortalidade, entre os grupos de categorias diferentes de FE e etiologias de IC. Quando a morte cardiovascular foi analisada, ICFEr, ICFEI, e fibrilação atrial foram identificados como fatores de risco ( Tabela 7 ).

**Tabela 7:** – Regressão logística multivariada e mortalidade cardiovascular

Regressão logística multivariada	p	Odds Ratio	Intervalo de confiança de 95%
ICFEr	0,003	2,23	1,13 - 3,78
ICFEI	0,034	2,04	1,06 - 4,08
ICFEp	_	1	_
Fibrillation atrial	0,004	2,31	1,31 - 4,08

*ICFEr: insuficiência cardíaca com fração de ejeção reduzida; ICFEI: insuficiência cardíaca com fração de ejeção intermediária; ICFEp: insuficiência cardíaca com fração de ejeção preservada.*

## Discussão

Existe um debate sobre qual o melhor método para avaliar o prognóstico em pacientes com IC, além da FE, levando-se em consideração a etiologia isquêmica, o remodelamento ventricular, comorbidades, entre outros.^7 , 17 , 18^ Sabe-se também que a FE é uma medida dinâmica, com uma variação intraobservador e interobservador de 7%, sendo possível reclassificar 80% dos pacientes com IC.^3 , 19 - 21^ Em sua última diretriz sobre IC de 2016, a Sociedade Europeia de Cardiologia recomenda identificar aqueles pacientes com ICFEI. Nas diretrizes de 2013 para o manejo da IC da *American Heart Association, American College of Cardiology* , e *Heart failure Society of America* , utilizam-se o termo “borderline” para pacientes com característics clínicas similares à ICFEp e “melhores” (improved) para pacientes isquêmicos com melhora na FE após evento agudo, mas ambos os termos como uma subclassificação da ICFEp. A atualização de 2017 não menciona uma nova classificação para a FE.^1^ A Sociedade Brasileira de Cardiologia, em sua diretriz mais recente sobre IC (2018), também adotou o termo ICFEI de uma maneira dinâmica, com uma prevalência de aproximadamente 10-20%, o que está de acordo com os nossos achados no presente estudo (prevalência de 17%).^3 , 7 , 18^

Em relação às características clínicas, os pacientes com ICFEI apresentam prevalência intermediária de comorbidades em comparação aos pacientes com ICFEr e ICFEp.^3 , 13 , 14 , 21^ A etiologia isquêmica parece ter prevalência similar em pacientes com ICFEI e paciente com ICFEr, em concordância com o presente estudo.^3 , 7 , 14 , 21^ No entanto, outros estudos relataram prevalência semelhante de comorbidades entre pacientes com ICFEI e ICFEp.^13 , 14^

O I Registro Brasileiro de IC Aguda (BREATHE), publicado em 2015, apresentou uma mortalidade hospitalar de 13%, ao passo que registros norte-americanos e europeus relatam uma taxa de mortalidade hospitalar de 4%. Esse dado indica diferenças importantes em relação à mortalidade hospitalar entre países desenvolvidos e em desenvolvimento. No presente estudo, a mortalidade hospitalar foi de 8%. Tal fato pode ser explicado pelo local do estudo, um hospital terciário, referência em cardiologia, equipado com uma unidade coronariana. Assim como no estudo BREATHE, a baixa adesão medicamentosa e infecção foram as principais causas de IC descompensada. Enquanto a primeira foi mais expressiva na população com ICFEr, a segunda mais comum na ICFEp. Pacientes com ICFEI apresentaram uma maior tendência a descompensar por isquemia miocárdica, o que pode explicar por que essa população apresentou maior etiologia isquêmica. Estudos recentes com pacientes com ICFEI aguda não investigaram a causa de descompensação.^13 , 14 , 16^

Há um entendimento clássico de que quanto maior a FE, maior a taxa de sobrevida, o que sugere um papel prognóstico importante da FE.^8^ Estudos recentes que analisaram a mortalidade em pacientes com ICFEI mostraram resultados conflitantes.^3 , 24 , 25^ Enquanto em alguns desses estudos não houve diferença na mortalidade geral entre os grupos,^10 , 13 , 14^ em outros, foram detectadas diferenças nas taxas de mortalidade entre ICFEr e ICFEp^7 , 8 , 21^ ou similar com pacientes com ICFEp.^12 , 16 , 20 , 23^ No presente estudo, não foi detectada diferença na mortalidade geral entre as três categorias de FE. Contudo, quando foram analisadas mortes cardiovasculares, os pacientes com ICFEI apresentaram pior prognóstico, similar aos pacientes com ICFEr. Isso pode ser explicado pelo fato que a maioria dos pacientes com ICEFI apresentavam isquemia miocárdica, um fator de pior prognóstico. Em nosso estudo, não conseguimos comprovar relação direta entre mortalidade e etiologia isquêmica por meio da regressão logística. Outra possível interferência é o impacto de comorbidades sobre mortes não cardiovasculares em pacientes com ICFEp.

Foi realizada regressão logística univariada para identificar o valor prognóstico de algumas características dos pacientes com IC em relação à mortalidade geral. Níveis elevados de ureia foram identificados como um fator de risco, e elevada pressão arterial identificada como um fator protetor. Esse dado está de acordo com o escore ADHERE ( *Acute Decompensated Heart Failure National Registry* ) em pacientes admitidos com IC aguda, que demonstrou pior prognóstico em pacientes com pressão sistólica inferior a 115 mmHg, níveis de creatinina maior que 2,75 mg/dL e de ureia maior que 92 mg/dL.^5^ A fibrilação atrial também foi um fator de risco nas análises univariada e multivariada, o que está de acordo com estudos prévios.^26 , 27^ Na análise multivariada de dados de mortalidade cardiovascular, pacientes com ICFEr e ICFEI apresentaram risco de morte duas vezes maior que pacientes com ICFEp, em conformidade com estudos recentes,^14 , 16^mas discordantes com estudos que não apresentaram diferença na mortalidade entre categorias de FE.^10 - 12 , 15^

O plano de ação global para a prevenção e controle de doenças crônicas não transmissíveis (2013-2020) da Organização Mundial de Saúde foi criado com a intenção de diminuir o impacto dessas doenças principalmente reduzindo-se os fatores de risco. Ao se comparar dados sobre doença cardiovascular e mortalidade, incluindo pacientes com IC, encontraram-se diferenças ao se comparar países desenvolvidos e em desenvolvimento.^28^ No Brasil, a IC é principalmente causada por doenças isquêmicas, hipertensivas e valvares, e ainda representa uma importante manifestação cardíaca da doença de Chagas e doenças reumáticas. Os recursos e a abordagem necessários aos pacientes com IC muitas vezes não são providos pelo sistema público de saúde local, causando um impacto negativo na hospitalização e mortalidade, como mostrado neste estudo, em comparação a países desenvolvidos. Estudos observacionais e registros têm se tornado extremamente importantes para ajudar a orientar estratégias de saúde pública de acordo com demandas e recursos locais.^29^ Em um estudo recente sobre ICEFI, os autores descreveram achados variados em relação a características clínicas e fenotípicas, e desfechos e tratamentos em pacientes com ICFEI, justificando a complexa análise dessa população. Esperamos que nosso estudo possa contribuir para o melhor entendimento dessa questão.^30^

### Limitações

A pequena amostra de 380 pacientes pode explicar o fato de o modelo regressão logística não ter sido capaz de mostrar significância estatística em relação a características importantes dos pacientes com IC. O estudo foi conduzido em um único centro de atenção terciária, referência em cardiologia, o que pode limitar a validação externa do estudo. Uma vez que a mortalidade foi verificada pelo Sistema de Informação sobre Mortalidade, podem ter ocorrido perdas de seguimento. Devido a dificuldades logísticas, nenhum dos pacientes foi contatado após a alta hospitalar para se verificar reinternação hospitalar, a qual é um importante desfecho.

## Conclusão

Não houve diferença na sobrevida geral entre os pacientes com ICFEr, ICFEI, e ICFEp. Pacientes com ICFEI e pacientes com ICFEr apresentaram maior mortalidade por doença cardiovascular em comparação a pacientes com ICFEp. A mortalidade hospitalar foi maior em comparação a de países desenvolvidos. Pacientes com ICFEI apresentaram características clínicas intermediárias às observadas entre as categorias de FE, e isquemia como principal causa da IC.

## References

[1] . Ponikowski P, Voors AA, Anker SD, Bueno H, Cleland JG, Coats AJ, et al. 2016 ESC Guidelines for the diagnosis and treatment of acute and chronic heart failure. Eur Heart J. Jul 2016;37(27):2129-200 10.1093/eurheartj/ehw128 27206819

[2] . Yancy CW, Jessup M, Bozkurt B, Butler J, Casey DE Jr, Drazner MH, et al. 2013 ACCF/AHA guideline for the management of heart failure. J Am Coll Cardiol. 2013 Oct 15;62(16):e147-239. 10.1016/j.jacc.2013.05.019 23747642

[3] . Rohde LE, Montera MW, Bocchi EA, Clausell NO, Albuquerque DC, Rassi S, et al. Brazilian Guideline for Chronic and Acute Heart Failure. Arq Bras Cardiol. 2018 Sep;111(3):436-539. 10.5935/abc.20180190 30379264

[4] . Albuquerque DC, Neto JDS, Bacal F, Rohde LE, Pereira SB, Berwanger O, et al. I Brazilian Registry of Heart Failure - Clinical Aspects, Care Quality and Hospitalization Outcomes. Arq Bras Cardiol. 2015 Jun;104(6):433-42. 10.5935/abc.20150031 PMC4484675 26131698

[5] . Adams KF Jr, Fonarow GC, Emerman CL, LeJemtel TH, Costanzo MR, Abraham WT, et al. ADHERE Scientific Advisory Committee and Investigators Characteristics and outcomes of patients hospitalized for heart failure in the United States: rationale, design, and preliminary observations from the first 100,000 cases in the Acute Decompensated. Heart Failure National Registry (ADHERE). Am Heart J. 2005 Feb;149(2):209-16. 10.1016/j.ahj.2004.08.005 15846257

[6] . Maggioni AP, Dahlström U, Filippatos G, Chioncel O, Crespo Leiro M, Drozdz J, et al. Heart Failure Association of the European Society of Cardiology (HFA), EURObservational Research Programme: regional differences and 1-year follow-up results of the Heart Failure Pilot Survey (ESC-HF Pilot). Eur J Heart Fail . 2013 Jul;15(7):808-17. 10.1093/eurjhf/hft050 23537547

[7] . Lam CSP, Solomon SD. The middle child in heart failure: heart failure with mid-range ejection fraction (40-50%), Editorial. Eur J Heart Fail. 2014 Oct;16(10):1049-55. 10.1002/ejhf.159 25210008

[8] . Meta-analysis Global Chronic Heart Failure (MAGGIC). The survival of patients with heart failure with preserved or reduced left ventricular ejection fraction: an individual patient data meta-analysis. Eur Heart J. 2012 Jul;33(14):1750-7. 10.1093/eurheartj/ehr254 21821849

[9] . Lunch LH. Heart Failure with “Mid-Range” Ejection Fraction – New Opportunities. J Cardiac Fail. 2016 Oct;22(10):769-71. 10.1016/j.cardfail.2016.07.439 27469481

[10] . Toma M, Ezekowitz JA, Bakal JA, O’Connor CM, Hernandez AF, Sardar MR, et al. The relationship between left ventricular ejection fraction and mortality in patients with acute heart failure: insights from the ASCEND-HF Trial. Eur J Heart Fail. 2014 Mar;16(3):334-41. 10.1002/ejhf.19 24464687

[11] . Gomez-Otero I, Ferrero-Gregori A, Roman AV, Amigo JS, Pascual-Figal DA, Jiménez JD, et al. Mid-range Ejection Fraction Does Not Permit Risk Stratification Among Patients Hospitalized for Heart Failure. Rev Esp Cardiol (Engl Ed). 2017 May;70(5):338-46. 10.1016/j.rec.2016.11.016 28011188

[12] . Rickenbacher P, Kaufmann BA, Maeder MT, Bernheim A, Goetschalckx K, Pfister O, et al. Heart failure with mid-range ejection fraction: a distinct clinical entity? Insights from the Trial of Intensified versus standard Medical therapy in Elderly patients with Congestive Heart Failure (TIME-CHF). Eur J Heart Fail. 2017 Dec;19(12):1586-96. 10.1002/ejhf.798 28295985

[13] . Chioncel O, Lainscak M, Seferovic PM, Anker SD, Crespo-Leiro MG, Harjola VP, et al. Epidemiology and one-year outcomes in patients with chronic heart failure and preserved, mid-range and reduced ejection fraction: an analysis of the ESC Heart Failure Long-Term Registry. Eur J Heart Fail. 2017 Dec;19(12):1574-85. 10.1002/ejhf.813 28386917

[14] . Takei M, Kohsaka S, Shiraishi Y, Kohno T, Fukuda K, Yoshikawa T, et al. Heart Failure with Mid-Range Ejection Fraction in Patients Admitted for Acute Decompensation: A Report from the Japanese Multicenter Registry. J Card Fail. 2019 Aug;25(8):666-73. 10.1016/j.cardfail.2019.05.010 31129270

[15] . Lam CS, Gamble GD, Ling LH, Sim D, Leong KT, Yeo PS, et al. Mortality associated with heart failure with preserved vs, reduced ejection fraction in a prospective international multi-ethnic cohort study. Eur Heart J. 2018 May 21;39(20):1770-80. 10.1093/eurheartj/ehy005 29390051

[16] . Farmakis D, Simitsis P, Vasiliki Bistola V, Triposkiadis F, Ikonomidis I, Katsanos S, et al. Acute heart failure with mid-range left ventricular ejection fraction: clinical profile, in-hospital management, and short-term outcome. Clin Res Cardiol. 2017 May;106(5):359-68. 10.1007/s00392-016-1063-0 27999929

[17] . Villacorta H, Mesquita ET. Prognostic Factors in Patients with Congestive Heart Failure. Arq Bras Cardiol. 1999;72(3):343-62. 10.1590/s0066-782x1999000300008 10513046

[18] . Get With The Guidelines - American Heart Association. [Cited in 2018 Jan 10], Available from: http://www,heart,org/HEARTORG/Professional/ GetWithTheGuidelines/Get-With-The-Guidelines---HFStroke_UCM_ 001099_ SubHomePage,jsp,

[19] . Felker GM, Shaw LK, O’Connor CM. A Standardized Definition of Ischemic Cardiomyopathy for Use in Clinical Research. J Am Coll Cardiol. 2002 Jan 16;39(2):210-8. 10.1016/s0735-1097(01)01738-7 11788209

[20] . Rastogi A, Novak E, Platts AE, Mann DL. Epidemiology, pathophysiology and clinical outcomes for heart failure patients with a mid-range ejection fraction. Eur J Heart Fail . 2017 Dec;19(12):1597-605. 10.1002/ejhf.879 PMC5730502 29024350

[21] . Tsuji K, Sakata Y, Nochioka K, Miura M, Yamauchi T, Onose T, et al. Characterization of heart failure patients with mid-range left ventricular ejection fraction-a report from the CHART-2 Study. Eur J Heart Fail. 2017 Oct;19(10):1258-69. 10.1002/ejhf.807 28370829

[22] . Yancy CW, Jessup M, Bozkurt B, Butler J, Casey DE Jr, Colvin MM, et al. 2017 ACC/AHA/HFSA Focused Update of the 2013 ACCF/AHA Guideline for the Management of Heart Failure: A Report of the American College of Cardiology/American Heart Association Task Force on Clinical Practice Guidelines and the Heart Failure Society of America. Circulation. 2017 Aug 8;136(6):e137-61. 10.1161/CIR.0000000000000509 28455343

[23] . Lopatin Y. Heart Failure with Mid-Range Ejection Fraction and How to Treat It. Card Fail Rev. 2018 May;4(1):9-13. 10.15420/cfr.2018:10:1 PMC5971675 29892469

[24] . Nauta JF, Hummel YM, vanMelle JP, van der Meer P, Lam CS, Ponikowski P, et al. What have we learned about heart failure with mid-range ejection fraction one year after its introduction? Eur J Heart Fail. 2017 Dec;19(12):1569-73. 10.1002/ejhf.1058 29067761

[25] . Gianluigi S, Vedin O, D’Amario D, Uijl A, Dahlström U, Rosano G, et al. Prevalence and Prognostic Implications of Longitudinal Ejection Fraction Change in Heart Failure. JACC Heart Fail. 2019 Apr;7(4):306-17. 10.1016/j.jchf.2018.11.019 30852236

[26] . Chamberlain AM, Redfield MM, Alonso A, Weston SA, Roger VL. Atrial fibrillation and mortality in heart failure: a community study. Circ Heart Fail. 2011 Nov;4(6):740-6. 10.1161/CIRCHEARTFAILURE.111.962688 PMC3222889 21920917

[27] . Piccini JP, Allen LA. Heart Failure Complicated by Atrial Fibrillation; Don’t Bury the Beta-Blockers Just Yet. JACC Heart Fail. 2017 Feb;5(2):107-9. 10.1016/j.jchf.2016.12.003 28089314

[28] . Global Burden of Disease Risk Factors Collaborators. Global, regional, and national comparative risk assessment of 79 behavioural, environmental and occupational, and metabolic risks or clusters of risks, 1990-2015: a systematic analysis for the Global Burden of Disease Study 2015. Lancet. 2016 Oct 8;388(10053):1659-724. 10.1016/S0140-6736(16)31679-8 PMC5388856 27733284

[29] . Bocchi EA. Heart Failure in South America. Curr Cardiol Rev. 2013 May; 9(2):147-56. 10.2174/1573403X11309020007 PMC3682398 23597301

[30] . Mesquita ET, Barbetta LMS, Correia ET. Heart Failure with Mid-Range Ejection Fraction – State of the Art. Arq Bras Cardiol. 2019; 112(6):784-90. 10.5935/abc.20190079 PMC6636372 31314831

