# Supplementary material for: Sobrevida de Pacientes com Insuficiência Cardíaca Aguda e Fração de Ejeção Intermediária em um País em Desenvolvimento – Estudo de Coorte no Sul do Brasil
Source: Arq Bras Cardiol. 2021 Jan 27;116(1):14–23. [Article in Portuguese] doi: 10.36660/abc.20190427 (PMC8159506; doi:10.36660/abc.20190427)
Supplement: Translation

# Translation: Survival of Patients with Acute Heart Failure and Mid-range Ejection Fraction in a Developing Country – A Cohort Study in South Brazil

**Authors:** Lucas Celia Petersen, Luiz Claudio Danzmann, Eduardo Bartholomay, Luiz Carlos Bodanese, Brenda Gonçalves Donay, Ellen Hettwer Magedanz, Adriana Vier Azevedo, Gustavo Farias Porciuncula, Marcelo Haertel Miglioranza

**Affiliations:** 1 Brazil Hospital São Lucas, Porto Alegre, RS - Brazil; 2 Brazil Hospital Universitário da Universidade Luterana do Brasil, Canoas, RS - Brazil; 3 Brazil Hospital Moinhos de Vento, Porto Alegre, RS - Brazil; 4 Brazil Instituto de Cardiologia do Rio Grande do Sul - Laboratório de Pesquisa e Inovação em Imagem Cardiovascular, Porto Alegre, RS - Brazil; 5 Brazil Prevencor - Hospital Mãe de Deus, Porto Alegre, RS - Brazil

**Keywords:** Survivorship, Heart Failure, Stroke Volume, Prognosis, Mortality, Medication Adherence, Epidemiology

## Abstract

**Background:**

Heart Failure with mid-range Ejection Fraction (HFmEF) was recently described by European and Brazilian guidelines on Heart Failure (HF). The ejection fraction (EF) is an important parameter to guide therapy and prognosis. Studies have shown conflicting results without representative data from developing countries.

**Objective:**

To analyze and compare survival rate in patients with HFmEF, HF patients with reduced EF (HFrEF), and HF patients with preserved EF (HFpEF), and to evaluate the clinical characteristics of these patients.

**Methods:**

A cohort study that included adult patients with acute HF admitted through the emergency department to a tertiary hospital, reference in cardiology, in south Brazil from 2009 to 2011. The sample was divided into three groups according to EF: reduced, mid-range and preserved. A Kaplan-Meier curve was analyzed according to the EF, and a logistic regression analysis was done. Statistical significance was established as p < 0.05.

**Results:**

A total of 380 patients were analyzed. Most patients had HFpEF (51%), followed by patients with HFrEF (32%) and HFmEF (17%). Patients with HFmEF showed intermediate characteristics related to age, blood pressure and ventricular diameters, and most patients were of ischemic etiology. Median follow-up time was 4.0 years. There was no statistical difference in overall survival or cardiovascular mortality (p=.0031) between the EF groups (reduced EF: 40.5% mortality; mid-range EF 39.7% and preserved EF 26%). Hospital mortality was 7.6%.

**Conclusion:**

There was no difference in overall survival rate between the EF groups. Patients with HFmEF showed higher mortality from cardiovascular diseases in comparison with HFpEF patients. (Arq Bras Cardiol. 2021; 116(1):14-23)

## Introduction

Heart Failure (HF) is a complex syndrome considered one of the major causes of hospital admission, morbidity, and mortality worldwide.^1 - 3^Observational studies have described mortality rates from HF ranging from 4% to 12% during hospitalization and 20% to 30% one year after discharge. Readmission rates are also high ranging from 20% to 30% in 90 days and up to 60% in one year.^3 - 6^Advances in cardiovascular therapy have been associated with a higher life expectancy and increased prevalence of HF in the elderly population, creating the need for a better knowledge of epidemiology, diagnosis and therapeutics of this important public health disease in developed and developing countries.

Although ejection fraction (EF) is not an ideal parameter to stratify HF patients, it has been historically used to guide therapy and determine prognosis in clinical practice.^7 , 8^To stimulate research and better categorize HF patients, the European Society of Cardiology created a new EF category in its recent guideline – HF with mid-range EF (HFmEF) – addressing patients with EF between 40-49%.^1^This new classification was also adopted by the Brazilian Society of Cardiology by the 2018 guideline on HF.^3^Since then, many studies have described the clinical outcomes and characteristics of the HFmEF population, with conflicting results.^9^While some studies with acute and chronic HF patients have shown similar survival among the three EF categories,^10 - 14^others have shown better survival of HFmEF and HF with preserved EF (HFpEF) as compared with HF patients with reduced EF (HFrEF).^15 , 16^

Data about HFmEF patients in Brazil and in developing countries are scarce in the literature. The objective of this study is to analyze survival and clinical characteristics of patients with HFmEF in comparison with patients admitted with acute HF (AHF) presenting reduced or preserved EF.

## Methods

### Study Design and Population

This was a prospective cohort study, derived from a clinical registry of 424 consecutive patients admitted with AHF to the emergency department of São Lucas hospital / Pontifícia Universidade Católica do Rio Grande do Sul, during the period from January 2009 to December 2011 ( Figure 1 ). The inclusion criteria were: 1) age above 18 years old; 2) AHF diagnosis defined by the Framingham criteria and later confirmed with transthoracic echocardiography. Patients who did not realize an echocardiography during the hospital stay were excluded. The clinical registry protocol was approved by the Research Ethics Committee of São Lucas Hospital (city of Porto Alegre) and a databank of AHF was developed. An informed consent form was obtained from participants.

**Figure 1:** – Study population with median follow-up of 4.0 years; HF: heart failure.

Sample size calculation was based on the Meta-Analysis Global Group in Chronic Heart Failure (MAGGIC), published in 2012. To observe a difference in mortality, it would be needed between 330 and 364 patients, with an 80% power and a 5% alpha error (Roasoft and WinPepi Sample Size Calculator Software).

### Clinical Assessment and Data Collection

Clinical assessment and treatment of patients included in the study were conducted by the emergency physician and the cardiology team on call according to the institutional routine protocol, without interference from the researchers. Data collection was done using a structured research form and medical chart reviews.

Patient’s initial signs and symptoms were registered at arrival to the emergency department by assessment of clinical status, hemodynamic profile, vital signs and New York Heart Association functional class, prior to admission. In addition to the treatment prescribed during the hospital stay, medications used at home and prescribed on discharge were also evaluated.

Causes of HF decompensation were analyzed: myocardial ischemia (if any type of myocardial revascularization was performed during hospital stay); uncontrolled hypertension (if hypertension stage ≥ II on arrival); arrhythmia (any non-sinus rhythm, except for permanent atrial fibrillation with controlled ventricular rate); poor medication adherence; infection (diagnosis during hospital stay).

Ischemic etiology of HF was considered when previous or recent myocardial revascularization was performed; functional test with ischemia higher than 10%; and anatomical examination revealing stenosis greater than 50% in the left main coronary artery or 70% in the proximal left anterior descending artery or other two coronary vessels. Self-reported comorbidities or those diagnosed during hospital stay were also registered.

As part of the institutional protocol, every patient underwent a 12-lead electrocardiography, chest radiography, laboratory exams (complete blood count, electrolytes, renal function, lipid profile, glucose, and urine analysis) and a transthoracic echocardiogram with measurement of EF by Simpson’s method.

The sample was divided in three groups according to left ventricular EF measured on echocardiogram: reduced (<40%), mid-range (40-49%) and preserved (≥ 50%). The diagnosis of HFpEF was made according to existing guidelines, based mainly on atrial diameter, left ventricular mass and diastolic function.

### Follow-up and Outcomes

Outcome data were obtained through medical chart review and through the Mortality Information System of the Health Center Information of the Rio Grande do Sul state to identify mortality and cause of death until December 2017.

Direct cause of death was established according to the International Classification of Diseases 10^th^edition.

The primary outcome assessed was overall mortality and secondary outcome was mortality from cardiovascular causes (acute myocardial infarction, HF, stroke, and arrhythmia).

### Statistical Analysis

Continuous variables with normal distribution (analyzed by the Kolmogorov-Smirnov test) were expressed as average and standard deviation or median and interquartile range, as appropriate. Comparison between categorical variables was performed by the chi-square test, and comparison between continuous variables was performed by analysis of variance (ANOVA) and Bonferroni post hoc test. Survival curves were estimated by the Kaplan-Meier method, using the log rank test statistics to compare EF categories. Univariate and multivariate logistic regression were assessed to determine the main variables related to mortality. Statistical significance was established with a p value < 0.05. Statistical analysis was performed using the Statistical Package for the Social Sciences (SPSS) Statistics, version 21.0.0.

## Results

Of 424 patients admitted with AHF, 380 patients were studied ( Figure 1 ). Most of patients had HFpEF (51.6%), followed by HFrEF (31.8%) and HFmEF (16.6%). Average age was 68 ±13 years old, mostly females (53%). The median follow up time was 4.0 years (interquartile range: 0.92 – 7.62 years).

### Clinical Characteristics

The patient population with HFpEF was mostly older women with higher levels of blood pressure and lower heart rate and left ventricle dimensions. The HFrEF group was mostly composed of young men with lower levels of blood pressure and higher heart rate and left ventricle dimensions. Patients with HFmEF presented intermediate characteristics between HFpEF and HFrEF population regarding to age, gender, blood pressure, heart rate and ventricle dimensions ( Tables 1 and 2 ).

**Table 1:** – Demographic data and comorbidities of patients with heart failure according to ejection fraction

Characteristics	Total	Ejection fraction < 40%	Ejection fraction 40-49%	Ejection fraction ≥ 50%	p
	**% (N = 380)**	**31.8% (N=121)**	**16.6% (N=63)**	**51.6% (N=196)**	
**Demographics**					
Age (mean in years)	68.1 ±13.8	64.0 ±12,6^b^	66.6 ±15,3^ab^	71.3 ±13,4^a^	<0.001
Female	52.9% (201)	35.5%(43)^b^	52.4%(33)^ab^	63.8%(125)^a^	<0.001
Body Mass Index (mean in Kg/m^2^)	28.1 ±6,5	26.6 ±6,1	29.2 ±6,3	28.6 ±6,6	0.100
**Comorbidities**					
Ischemic etiology	40.0% (152)	46.3% (56)	52.4% (33)	32,1% (63)	0.004
Hypertension	93.2% (354)	90.1% (109)	92.1% (58)	95.4% (187)	0.176
Dyslipidemia	74.8% (243)	76.2% (80)	76.9% (40)	73.2% (123)	0.796
Chronic Renal Disease	46.2% (156)	42.1% (45)	57.9% (33)	44.8% (78)	0.135
Diabetes Mellitus	45.9% (169)	43.9% (50)	50.8% (32)	45.5% (87)	0.668
Valvulopathy	35.1% (99)	28,1% (25)	36.2% (17)	39.0% (57)	0.230
Chronic Obstructive Pulmonary Disease	32.2% (111)	42.1%(45)	21.1% (12)	29.8% (54)	0.014
Implantable Cardiac Device	20.7% (78)	27.3% (33)	24.6% (15)	15.5% (30)	0.031
Atrial Fibrillation	20.0% (76)	5.8% (22)	2.1% (8)	12.1% (42)	0.085
Left Bundle Branch Block	16.3% (62)	7.1% (27)	2.9% (11)	6.3% (24)	0.133
Stroke	17.5% (62)	16.2% (18)	12.3% (7)	19.8% (37)	0.390
Hypothyroidism	18.0% (49)	16.7% (14)	28.9% (13)	15.4% (22)	0.112
Alcohol abuse	19.4% (67)	32.4% (34)	12.5% (7)	14.1% (26)	<0.001
Smoking	17.7% (63)	25.9% (29)	11.9% (7)	14.6% (27)	0.021
Cancer	12.0% (43)	12.4% (14)	3.4% (2)	14.5% (27)	0.070

*Statistical analysis: Chi-square test with adjusted residual and ANOVA with Bonferroni test when applied (small letters a and b).*

**Table 2:** – Clinical, laboratory and image data on admission

Characteristics	Total	Ejection fraction < 40%	Ejection fraction40-49%	Ejection fraction ≥ 50%	p
	**% (N = 380)**	**31.8% (N=121)**	**16.6% (N=63)**	**51.6% (N=196)**	
**Demographics**					
Systolic Blood Pressure (mean in mmHg)	140 (±35)	128 (±26)b	139 (±33)ab	147 (±39)a	<0.001
Heart Rate (mean in bpm)	91 (±23)	96 (±22)a	89 (±20)ab	88 (±22)b	0.006
Hemoglobin (mg/mL)	12.0 (±2.6)	12.6 (±2.5)a	11.9 (±2.3)ab	11.6 (±2.6)b	0.004
Creatinine (mg/dL)	1.8 (±1.2)	1.9 (±1.5)	1.9 (±0.9)	1.8 (±1.2)	0.615
Urea (mg/dL)	71 (±46)	70 (±48)	76 (±40)	71 (±50)	0.766
Sodium (mg/dL)	137 (±17)	139 (±4.4)	139 (±3.1)	137 (±2.5)	0.324
Potassium (mg/dL)	4.3 (±0.7)	4.4 (±0.8)ab	4.5 (±0.6)a	4.2 (±0.7)b	0.017
Left Ventricle Systolic Diameter (cm)	3.5 (±1.8)	5.0 (±1.6)a	4.0(±1.5)b	3.1 (±0.8)c	<0.001
Left Ventricle Diastolic Diameter (cm)	4.7 (±2.0)	5.7 (±1.8)a	5.2 (±1.9)b	4.8 (±0.9)b	<0.001
Left Atrium Diameter (cm)	3.9 (±1.7)	4.3 (±1.3)	4.0 (±1.5)	4.3 (±0.9)	0.182

*Statistical analysis: ANOVA test - with Bonferroni test when applied (small letters a, b and c).*

In the population with HFmEF patients, plasma potassium levels were higher at admission and myocardial ischemia was the main HF etiology ( Table 1 ). Patients with HFmEF had a smaller prevalence of chronic obstructive pulmonary disease, tobacco and alcohol use. Patients with HFrEF had a higher use of angiotensin converting enzyme inhibitor, antimineralocorticoid, digoxin and loop diuretics, and more implantable electronic cardiac devices ( Tables 2 and 3 ). Most patients presented with a “wet and warm” hemodynamic profile on admission, with no difference between the EF groups.

**Table 3:** – Medications at home

Medications	Total	EF < 40%	EF 40-49%	EF ≥ 50%	p
	**% (N = 380)**	**31.8% (N=121)**	**16.6% (N=63)**	**51.6% (N=196)**	
Loop diuretic	60.1% (218)	67.0% (77)	66.7% (38)	53.9% (103)	0.043
Angiotensin converting enzyme inhibitor	51.5% (187)	63.5% (73)	38.6% (22)	48.2% (92)	0.043
Betablocker	49.0% (179)	50.0% (58)	45.6% (26)	49.5% (95)	0.641
Acetylsalicylic Acid	40.7% (149)	44.0% (51)	45.6% (26)	37.3% (72)	0.367
Statin	43.3 (156)	43.0% (49)	50.0% (28)	41.6% (79)	0.533
Digoxin	25.6% (93)	40.0% (46)	24.6% (14)	17.3% (33)	<0.001
Oral antidiabetic	20.9% (76)	19.1% (22)	17.5% (10)	23.0% (44)	0.568
Insulin	19.3% (70)	20.9% (24)	24.6% (14)	16.8% (32)	0.370
Mineralocorticoid Receptor Antagonist	18.5% (67)	27.0% (31)	22.8% (13)	12.0% (23)	0.003
Calcium Channel Blocker	16.9% (61)	8.8% (10)	15.8% (9)	22.1% (42)	0.011
Thiazide Diuretic	14.6% (53)	14.0% (16)	14.0% (8)	15.2% (29)	0.954
Oral anticoagulation	14.0% (51)	14.7% (18)	10.5% (6)	14.1% (27)	0.660
Angiotensin Receptor Blocker	12.2% (44)	5.2% (6)	17.5% (10)	14.7% (28)	0.019

*Statistical analysis: Chi-Square test with adjusted residual.*

Poor adherence to medical therapy was the main cause of HF decompensation, followed by infection in patients with HFrEF and HFpEF respectively ( Table 4 ).

**Table 4:** – Causes of decompensation

Characteristics	Total	EF < 40%	EF 40-49%	EF ≥ 50%	p
	**% (N = 380)**	**31.8% (N=121)**	**16.6% (N=63)**	**51.6% (N=196)**	
**Causes of decompensation**					
Medications	30.5% (116)	42.1% (51)	27.0% (17)	24.5% (48)	0.003
Infection	27.1% (103)	19.0% (23)	19.0% (12)	34.7% (68)	0.003
Arrhythmia	18.7% (71)	15.7% (19)	19.0% (12)	20.4% (40)	0.721
Hypertension	14.5% (55)	9.1% (11)	15.9% (10)	17.3% (34)	0.120
Myocardial Ischemia	7.6% (29)	8.3% (10)	12.7% (8)	5.6% (11)	0.174
Salt overload	7.4% (28)	7.4% (9)	7.9% (5)	7.1% (14)	0.978
Unknown	18.2% (69)	18.2% (22)	23.8% (15)	16.3% (32)	0.407

*Statistical analysis: Chi-square test with adjusted residual.*

### Outcomes

In-hospital mortality was 7.6%. Overall mortality in the eight years of follow- up was 60.7%, with no significant difference between the EF categories ( Figure 2 ).

**Figure 2:** – Overall survival curve. HFrEF (heart failure with reduced ejection fraction); HFmrEF (heart failure with mid-range ejection fraction); HFpEF (heart failure with preserved ejection fraction).,

Mortality in the EF groups through the follow-up time is described in Table 5 .

**Table 5:** – Mortality during study follow up

Overall Mortality	Total	EF < 40%	EF 40-49%	EF ≥ 50%	p
	**% (N = 380)**	**31.8% (N=121)**	**16.6% (N=63)**	**51.6% (N=196)**	
In Hospital	7.6% (29)	6.6% (8)	4.8% (3)	9.2% (18)	0.453
1 month	10.8% (41)	10.7% (13)	7.9% (5)	11.7% (23)	0.699
3 months	14.7% (56)	13.2% (16)	14.3% (9)	15.8% (31)	0.814
12 months	26.6% (101)	28.5% (35)	27.0% (17)	25.0% (49)	0.742
5 years	55.0% (209)	60.3% (73)	52.4% (33)	52.6% (103)	0.439
8 years	60.7% (231)	65.3% (79)	63.5% (40)	57.1% (112)	0.398

*Statistical analysis: ANOVA test.*

Mean survival rate was 4.7 years (CI 95%: 3.7 – 5.6), with the tendency of a gradual increase with the EF (reduced EF: 4.3 years; mid-range EF: 4.7 years; and preserved EF: 4.9 years). Cardiovascular mortality was responsible for nearly half of the deaths (54.1%). There was a statistically significant difference between the EF groups when cardiovascular deaths were analyzed separately (p=0.031) – reduced EF: 40.5%; mid-range EF: 39.7%; and preserved EF: 26% ( Figure 3 ).

**Figure 3:** – Survival curve for cardiovascular cause. HFrEF (heart failure with reduced ejection fraction); HFmrEF (heart failure with mid-range ejection fraction); HFpEF (heart failure with preserved ejection fraction).

### Univariate Analysis

When univariate logistic regression was analyzed with categorical variables, the presence of atrial fibrillation and urea levels higher than 92 mg/dL were identified as risk factors. When analyzed as a continuous variable, higher values of systolic blood pressure were identified as a protective factor. Data collected at arrival to the emergency department are described in Table 6 .

**Table 6:** – Univariate logistic regression in relation to overall mortality

Univariate Logistic Regression	p	Odds Ratio	Confidence Interval 95%
HFrEF	0.245	1.44	0.78 - 2.65
HFmEF	0.62	1.2	0.58 - 2.49
HFpEF	_	1	_
Ischemic Etiology	0.775	1.07	0.66 - 1.74
Diastolic diameter > 5.6 cm	0.421	1.26	0.72 - 2.12
Systolic Blood Pressure < 115 mmHg	0.494	1.22	0.69 - 2.12
Systolic Blood Pressure	0.006	0.99	0.98 - 0.99
Creatinine > 2.75 mg/dl	0.741	1.15	0.51 - 2.58
Urea > 92 mg/dl	0.034	2.00	1.05 - 3.80
Atrial fibrillation	0.028	1.98	1.08 - 3.64
Left bundle branch block	0.921	1.03	0.54 - 1.97

*HFrEF: heart failure with reduced ejection fraction; HFmrEF: heart failure with mid-range ejection fraction; HFpEF: heart failure with preserved ejection fraction.*

### Multivariate Analysis

Multivariate logistic regression revealed that there was no difference in clinical characteristics or mortality rate between the groups of EF categories and HF etiologies. When cardiovascular death was analyzed, HFrEF, HFmrEF and atrial fibrillation were identified as risk factors ( Table 7 ).

**Table 7:** – Multivariate logistic regression and cardiovascular mortality

Multivariate Logistic Regression	p	Odds Ratio	Confidence Interval 95%
HFrEF	0.003	2.23	1.13 - 3.78
HFmEF	0.034	2.04	1.06 - 4.08
HFpEF	_	1	_
Atrial Fibrillation	0.004	2.31	1.31 - 4.08

*HFrEF (heart failure with reduced ejection fraction); HFmrEF (heart failure with mid-range ejection fraction); HFpEF (heart failure with preserved ejection fraction).*

## Discussion

There is a debate about how to better evaluate the prognosis in HF patients beyond EF, also considering ischemic etiology, ventricular remodeling, comorbidities, among others.^7 , 17 , 18^It is also known that EF is a dynamic measure with an intra- and inter-observer variability of 7%, making it possible to reclassify 80% of the HF patients.^3 , 19 - 21^In its last 2016 guidelines on HF, the European Society of Cardiology recommends identifying those patients with HFmEF. The American Heart Association / American College of Cardiology / Heart failure Society of America, in the 2013 guideline for the management of HF, use the term “borderline” for patients with clinical characteristics similar to HFpEF, and “improved” for ischemic patients with improved EF after the acute event, but both as HFpEF subclassification. The focused 2017 update does not mention a new EF classification.^1^The Brazilian Society of Cardiology in its latest 2018 HF guideline, also adopted the term HFmEF in a dynamic manner, with a prevalence of approximately 10-20%, in agreement with the 17% prevalence in the present study.^3 , 7 , 18^

In regard to clinical characteristics, patients with HFmEF have intermediate prevalence of comorbidities in relation to HFrEF and HFpEF patients.^3 , 13 , 14 , 21^The prevalence of ischemic etiology seems to be similar in HFmEF and HFrEF patients, in agreement with the present study.^3 , 7 , 14 , 21^However, other studies have reported similar prevalence of comorbidities between patients with HFmEF and HFpEF.^13 , 14^

The I Brazilian Registry of Acute Heart Failure (BREATHE) published in 2015 showed a hospital mortality of 13%, while American and European registries have reported 4% hospital mortality rate. This data indicates important differences regarding in-hospital mortality between developed and developing countries. In the present study, in-hospital mortality was 8%. This may be explained by the place of the study, a tertiary care hospital, reference in cardiology, with a coronary care unit. As in the BREATHE study, poor medication adherence and infection were the main causes of HF decompensation. The first was more representative in the HFrEF population, while the second in the HFpEF. Patients with HFmEF had a higher tendency to decompensate due to myocardial ischemia, which may explain why this population had a higher ischemic etiology. Recent studies with acute HFmEF patients did not investigate the cause of decompensation.^13 , 14 , 16^

There is a classical understanding that the higher the EF, the higher the survival rate, supporting an important prognostic role of EF.^8^Recent studies that analyzed mortality in HFmEF patients showed conflicting results.^3 , 24 , 25^In some of these studies, there was no difference in overall mortality between the groups,^10 , 13 , 14^while in others, showed mortality rates between HFrEF and HFpEF^7 , 8 , 21^or similar with HFpEF patients.^12 , 16 , 20 , 23^In the present study, there was no difference in overall mortality between the three EF categories. However, when cardiovascular deaths were analyzed, patients with HFmEF had a worse prognosis, similar to HFrEF patients. This may be explained by the fact that most of HFmEF patients had myocardial ischemia, a poor prognostic factor.^17^In our study, we were unable to proof a direct relation between mortality related to ischemic etiology through logistic regression. Another possible interference is the impact of comorbidities on non-cardiovascular deaths in HFpEF patients.

Univariate logistic regression was made to identify the prognostic value of some characteristics of HF patients regarding overall mortality. An elevated level of urea was identified as a risk factor and a higher blood pressure was identified as a protective factor. This data agrees with the ADHERE score (Acute Decompensated Heart Failure National Registry) in patients admitted with acute heart failure that demonstrated worse prognosis in patients with systolic blood pressure below 115mmHg, levels of creatinine above 2.75 mg/dL and urea above 92 mg/dL.^5^Atrial fibrillation was also a risk factor in the univariate and multivariate analysis, which also agrees with previous studies.^26 , 27^In the multivariate analysis with cardiovascular mortality data, HFrEF and HFmEF showed a twofold mortality risk when compared with HFpEF patients in agreement with recent studies,^14 , 16^but in discordance with studies that did not show a difference in mortality between EF categories.^10 - 12 , 15^

The ‘Global action plan for the prevention and control of noncommunicable diseases 2013-2020’ was created by the World Health Organization with the intention to reduce the impact of these diseases manly by risk factor reduction. When comparing data on cardiovascular disease and mortality, including HF patients, there have been differences when comparing developed and developing countries.^28^In Brazil, HF is mainly caused by ischemic, hypertensive and valve diseases, and still represent an important cardiac manifestation of Chagas disease and rheumatic disorders. The resources and management required by HF patients that are often not met by local public health systems, causing negative impact on hospitalization and mortality, as shown in this study, when compared with developed countries. Observational studies and registries become extremely important to help guide effective public health strategies according to local demands and resources.^29^In a recent ‘state of the art’ study about HFmEF, the authors reported various findings regarding clinical characteristics and phenotypes, and outcomes and treatment in patients with HFmEF, justifying the complex analysis of this patient population. We hope that our study can add to a better understanding of this issue.^30^

### Limitations

The small sample of 380 patients may explain the fact that the logistic regression model was not able to show statistical significance about important characteristics of HF patients. The study was conducted in a single tertiary center, reference in cardiology, which may limit the external validation of the study. As mortality was verified through the Mortality Information System, losses to follow-up may have occurred. Due to logistic difficulties, no contact was made with any of the patients after hospital discharge to verify readmission, an important outcome.

## Conclusion

There was no difference in overall survival between HF patients with reduced, intermediate, and preserved EF. HFmEF and HFrEF patients had a higher mortality from cardiovascular cause when compared with HFpEF patients. Hospital mortality was higher when compared with developed countries. HFmEF patients had clinical characteristics intermediate between EF categories, and ischemia as the main cause of HF.
